# Correction of haemorrhagic shock-associated coagulopathy and impaired haemostasis by plasma, prothrombin complex concentrates or an activated protein C-targeted DNA aptamer in mice

**DOI:** 10.1038/s41598-023-30794-7

**Published:** 2023-03-07

**Authors:** Louise J. Eltringham-Smith, Scott C. Meixner, Edward L. G. Pryzdial, William P. Sheffield

**Affiliations:** 1 Medical Affairs and Innovation, Canadian Blood Services, Hamilton, ON Canada; 2 Medical Affairs and Innovation, Canadian Blood Services, Vancouver, BC Canada; 3 Centre for Blood Research, University of British Columbia, Vancouver, BC Canada; 4 Department of Pathology and Molecular Medicine, McMaster University, HSC 4N66, 1280 Main Street West, Hamilton, ON L8S 4K1 Canada

**Keywords:** Biochemistry, Biological techniques, Drug discovery, Cardiology, Molecular medicine

## Abstract

Even with extensive transfusion support, trauma-induced bleeding often leads to death. Early intervention may improve outcomes, yet which blood products, factor concentrates, or other drugs constitute optimal treatment is unclear. Patients with acute traumatic coagulopathy (ATC), arising from trauma and haemorrhagic shock, have the worst prognosis. Here, multiple interventions were compared in a mouse model of ATC. After the trauma of tissue excision, anaesthetized mice were bled to 35 mm Hg mean arterial pressure, maintained under shock for 60 min, and resuscitated with fluids equal in volume to the shed blood. Resuscitated mice were subjected to liver laceration to test haemostasis and blood loss was quantified. Saline-treated mice lost two- to three-fold more blood than sham-treated animals and were coagulopathic by prothrombin time elevation post- versus pre-procedure. Murine fresh-frozen plasma (mFFP), anti-activated protein C aptamer HS02-52G, or prothrombin complex concentrates eliminated the bleeding diathesis and coagulopathy; fibrinogen, plasminogen activator inhibitor-1, or tranexamic acid ameliorated bleeding or coagulopathy, but not both. HS02-52G and mFFP also eliminated the changes in plasma aPC and tissue plasminogen activator levels observed in saline-treated mice, as judged via microtiter plate biomarker assays. Procoagulant interventions, especially inhibiting aPC, could be beneficial in human ATC.

## Introduction

Physical trauma results in a heavy burden of death and disability in both developing and developed countries. In 2021, the World Health Organization estimated that 4.4 million annual deaths arose from unintentional injury or violence worldwide^1^. Among these trauma patients, bleeding is the leading cause of preventable death^2^; its prevention likely requires extensive transfusion support for which optimal protocols are yet to be established^3,4^.

Evidence favours balanced transfusion support for critically injured patients admitted to trauma care hospitals^5^, often delivered via massive transfusion protocols (MTP)^6,7^. MTP feature early identification of massively bleeding patients and the rapid administration of pre-specified ratios of universal blood products in pre-specified or viscoelastometric-driven ratios^8,9^.

Early prehospital administration of plasma to trauma patients may also be beneficial, although available evidence is conflicting. Two randomized clinical trials (RCT) of prehospital plasma versus standard of care yielded conflicting results, with the Prehospital Air Medical Plasma (PAMPer) trial showing a statistically significant reduction in 30-day mortality but the Control of Major Bleeding After Trauma (COMBAT) trial finding no such benefit^10,11^. Another RCT, Resuscitation with pre-hospital blood products (RePHILL), found no survival benefit of red cell concentrates and plasma versus saline as prehospital interventions^12^. It remains unclear what blood products, plasma protein products, or novel or combination therapies would best address the unmet clinical need to improve outcomes in trauma.

Coagulopathy can worsen the prognosis of bleeding trauma patients when combined tissue injury and shock prompt hemostatic failure, in a complex disorder called acute traumatic coagulopathy (ATC) or trauma-induced coagulopathy (TIC)^13–18^. Some investigators separate TIC into an early hypocoagulable phase and a later hypercoagulable phase^19^. Given the challenges of clinical investigations in trauma^20^, many investigators have turned to animal models of ATC/TIC to gain mechanistic understanding and to test potential interventions^21^. Previously, we induced experimental coagulopathy and bleeding diathesis without shock by sequential blood exchange in mice, finding that procoagulant treatments (e.g. plasma, prothrombin complex concentrates, or purified prothrombin) restored haemostatic control^22,23^. In the current study, we adapted a previously described model of murine pressure-defined haemorrhagic shock and investigated the ability of different resuscitation fluids to restore haemostasis. Our hypothesis was that procoagulant fluid treatments would restore haemostatic control and ameliorate coagulopathy. Our objectives were to survey different resuscitation fluids, such as crystalloid, plasma, plasma protein concentrates, and procoagulant or anti-fibrinolytic agents, for their abilities to reverse coagulopathy and restore haemostasis in murine ATC/TIC, and to investigate their potential mechanisms of action. We report that murine plasma, human prothrombin complex concentrates, or a DNA aptamer that inhibits activated protein C (aPC)^24^ return bleeding levels to normal in ATC/TIC model mice and eliminate coagulopathy, while other treatments control one or the other aspect of ATC/TIC, but not both.

## Results

### General approach

Haemorrhagic Shock with Liver Laceration (HS/LL) is a model that combines elements of traumatic injury and hemorrhagic shock with fluid resuscitation and hemostatic challenge. The general conceptual approach is depicted in Fig. 1a, with a timeline of specific actions in Fig. 1b (see Methods).

**Figure 1:**
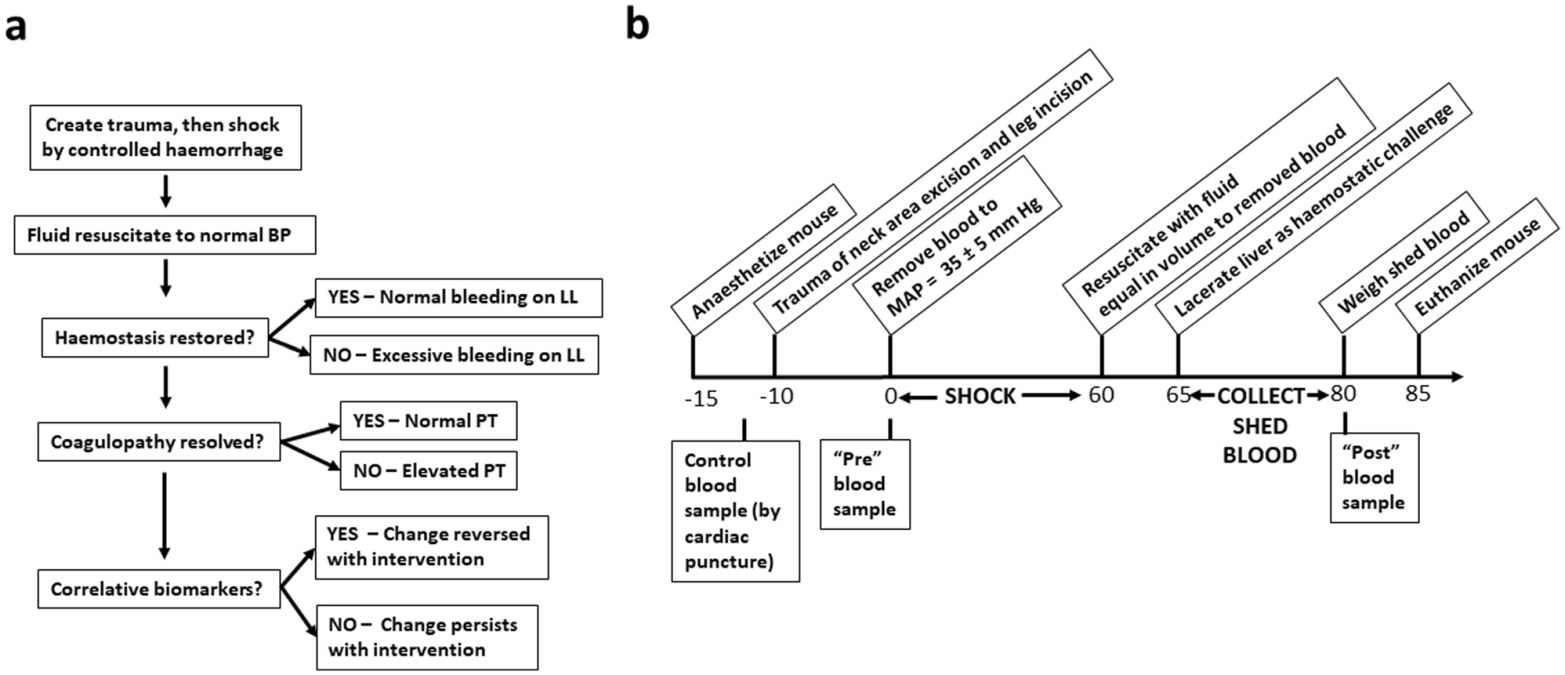
Mouse haemorrhagic shock model. (**a**) Overview of experimental design, measuring haemostasis by liver laceration (LL) and coagulopathy by prothrombin time (PT). (**b**) Experimental timeline initiated by anaesthetization of mice 15 min before induction of shock. Five minutes later they were subjected to minor trauma in placing a blood pressure probe in the femoral artery and in accessing and cannulating the carotid artery. Blood was drawn via the carotid cannula until the mean arterial pressure (MAP) reached a target of 35 ± 5 mM Hg. Mice remained in shock for 60 min and were then resuscitated to starting MAP by infusion of different resuscitation fluids equal in volume to the drawn blood, over 2- to 3- minutes, via the carotid cannula. Following a standardized liver injury, shed blood was collected for 15 min, then a final blood sample was taken from the carotid artery and the mouse was euthanized by exsanguination followed by cervical dislocation. “Pre” and “post” blood samples were processed to obtain plasma that was frozen for subsequent analysis. In some experiments control blood samples were taken from anaesthetized mice via cardiac puncture with no other treatment or intervention (Control) or from anaesthetized mice subjected to tissue trauma and cannulation only (Sham).

### Trauma-induced coagulopathy

Prothrombin time (PT) was measured in plasma from blood samples from control anaesthetized mice obtained by cardiac puncture, or in plasma from blood samples from cannulated sham-treated mice subjected to tissue trauma (surgical excision of neck area skin flap and incision to place pressure probe in femoral artery) but not haemorrhagic shock or resuscitation. PT values were significantly elevated in Sham versus Control samples by 40.4% (Fig. 2a).

**Figure 2:**
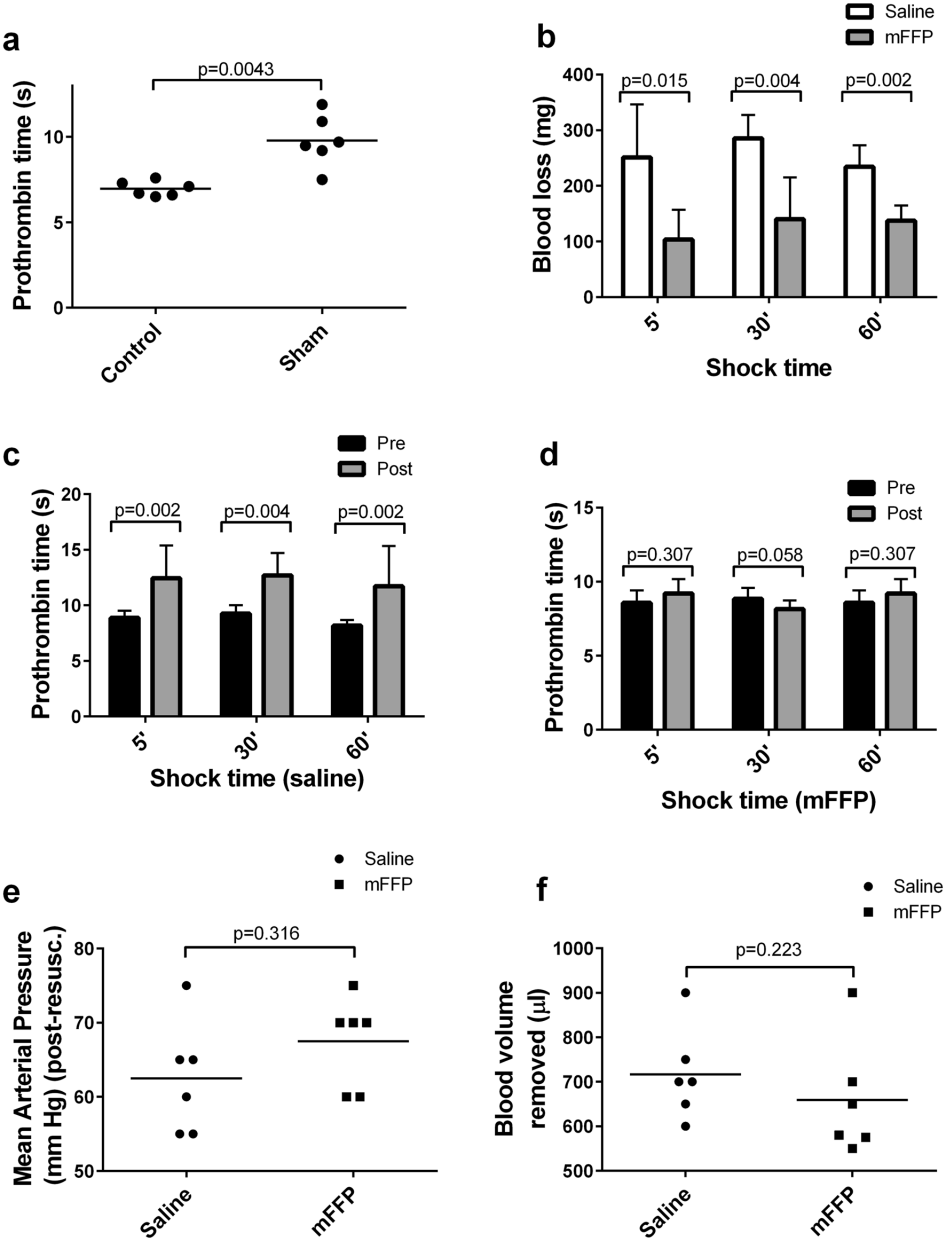
Comparison of saline and murine FFP as resuscitation fluids following various shock periods. (**a**) prothrombin time (PT) was determined for plasma from blood samples obtained by cardiac puncture from mice without any other treatment (Control) or for plasma from blood samples obtained via the carotid cannula of mice subjected to tissue injury and cannulation but not haemorrhage or shock (Sham). (**b**) mice were subjected to haemorrhagic shock for 5, 30, or 60 min and then resuscitated with saline (white bars) or murine FFP (mFFP, grey bars) and shed blood was weighed after liver laceration. (**c**) prothrombin times were determined for plasma from saline-treated mice mouse plasma drawn before shock (Pre, black bars) or after shock and resuscitation (Post, grey bars). (**d**) as in panel b but for mFFP resuscitated mice. **e**, post-resuscitation MAP (mm Hg) for mice subjected to 60 min shock and resuscitated with saline (white bars) or mFFP (grey bars). (**f**) as in panel e but for blood volume removed per mouse (µl) to achieve target MAP. All bars show the mean of 6 determinations ± SD. Horizontal bars give p values for data sets compared by Mann–Whitney U tests (**a**, **e**, **f**).

### Shock time

The time mice remained in shock was varied, in experiments following the same timeline as shown in Fig. 1B, except for the shock period. Mean blood losses were significantly reduced following liver laceration, by two- to three-fold, in mice resuscitated with mouse fresh-frozen plasma (mFFP), compared to those resuscitated with saline, whether the period of shock was 5, 30, or 60 min (Fig. 2b). Groups of mice resuscitated with saline were also uniformly coagulopathic, with significantly elevated pre- versus post-resuscitation PT values for all shock times (Fig. 2c). No such elevation was apparent in the plasma of mice resuscitated with mFFP (Fig. 2d). There was no significant difference in post-resuscitation mean arterial pressure (MAP) between mice subjected to 60 min of HS and resuscitated with either saline or mFFP (Fig. 2e) or in the mean blood volume removed to create the desired level of shock (35 ± 5 mM Hg) in either group (Fig. 2f). Based on the results of these pilot experiments, the shock time was therefore fixed at 60 min for all subsequent experiments in this study.

### Haemostasis following fluid resuscitation with DNA-free agents

Blood loss after haemostatic LL challenge following fluid resuscitation with different agents is shown in Fig. 3. Sham-treated mice lost 90 ± 40 mg of shed blood. Following HS and saline resuscitation, these losses rose significantly, by 2.7-fold, to 240 ± 40 mg. Similarly, elevated blood losses not differing significantly from saline were observed in mice treated with 5% human albumin solution (HAS) or purified human Plasminogen Activator Inhibitor-1 (PAI-1) at 40 µg/kg. In contrast, treatment with mFFP, purified human fibrinogen (140 mg/kg), human prothrombin complex concentrates (PCC, 14.3 IU/kg), or tranexamic acid (TXA, 150 mg/kg) significantly reduced blood losses versus saline, to levels not differing statistically from sham.

**Figure 3:**
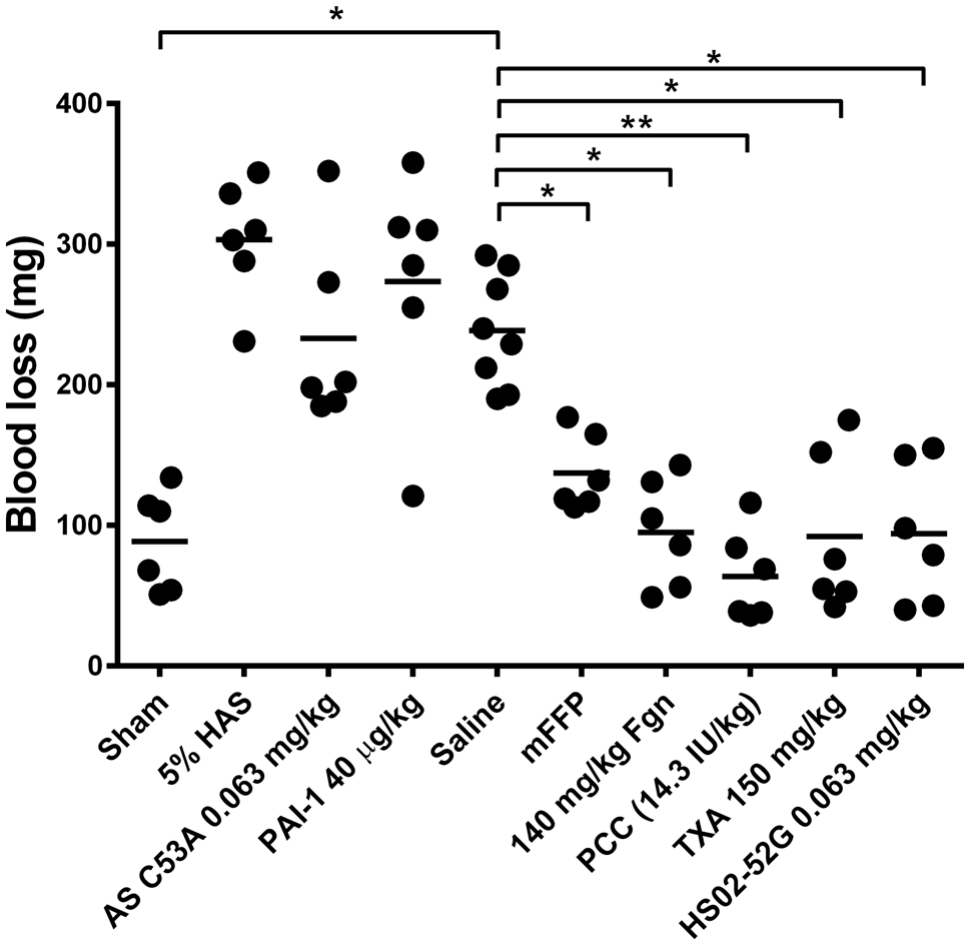
Blood loss following trauma, shock, resuscitation, and liver laceration in mice treated with different fluids. HS/LL mice were subjected to 60 min haemorrhagic shock (HS), then resuscitated with the resuscitation fluids and doses identified below the x-axis of each graph, equal in volume to the shed blood, prior to haemostatic challenge by liver laceration (LL). Sham mice were anaesthetized and cannulated but were not subjected to haemorrhagic shock, resuscitation, or liver laceration, but were otherwise maintained identically to protocol mice with respect to timing of sampling and handling. Each point represents a single mouse (n = 6 in all cases except n = 8 for saline). Horizontal lines within the scatter plots represent the mean of each group. Asterisks above horizontal brackets refer to statistical differences versus Saline between groups by Kruskal–Wallis tests with Dunn’s post-tests (*p < 0.05, **p < 0.01).

### Haemostasis following resuscitation with DNA-based agents

HS02-52G is a DNA aptamer previously shown to inhibit aPC in vitro^24^. Before employing this agent in vivo, we replicated its previously reported in vitro activity (Supplementary Fig. S1). Activated partial thrombin time (APTT) clotting assays supplemented with aPC were prolonged ~ two-fold, a prolongation reversed by inclusion of 100 nM HS02-52G but not unrelated oligonucleotide AS C53A; neither DNA molecule affected the APTT in the absence of added aPC (Supplementary Fig. S1A). HS02-52G reversed the aPC-mediated APTT prolongation in a dose-dependent manner; the effect of spiking 0.9 nM aPC into the APTT was eliminated at HS02-52G concentrations > 50 nM (Supplementary Fig. 1B). As shown in Fig. 3, resuscitation of HS mice with 63 µg/kg control aptamer AS C53A, a dose selected to deliver 100 nM DNA aptamer to the murine plasma compartment, was associated with blood losses that did not differ from saline treatment (230 ± 70 mg); in contrast, resuscitation with 63 µg/kg anti-aPC aptamer HS02-52G restored haemostatic control to levels not statistically different from sham (90 ± 50 mg).

### Post-treatment mean arterial pressure (MAP)

The MAP after resuscitation was compared for six of the treatment groups assessed in Fig. 3. Despite the likely difference in oncotic pressure among the treatment fluids (mFFP, HS02-52G, AS C53A, 14.3 IU/kg PCC, 140 mg/kg fibrinogen, and 40 µg/kg PAI-1), MAP after resuscitation did not differ statistically among the groups (see Supplementary Figure S2).

### Mortality

The HS/LL model was not, in general, associated with mortality during the experimental period, with 96.6% of mice (145/150 in total in this study) surviving to the euthanasia endpoint (see Materials and Methods for additional details).

### Clotting times before and after HS/LL

PT values were measured in plasma samples from the same mice whose blood losses are shown in Fig. 4, to compare clotting function between samples taken before the shock period (pre-HS/LL) and after HS/LL (post-HS/LL). Mean PT values were significantly elevated, by 18–44%, in post-HS/LL versus pre-HS/LL samples from mice resuscitated with saline, 140 mg/kg fibrinogen, 150 mg/kg TXA, or 63 µg/kg AS C53A (Fig. 4). In contrast, no PT-defined coagulopathy was observed in mice treated with mFFP, 14.3 IU/kg PCC, 40 µg/kg PAI-1, or 63 µg/kg HS02-52G. The high variance in post-procedure samples from mice treated with 140 mg/kg fibrinogen (21 ± 16 s) caused us to re-examine additional stored aliquots of plasma from this cohort. In some samples on close examination small aggregates were detected that may have in part contributed to the unusual results in this cohort not seen in any other group.

**Figure 4:**
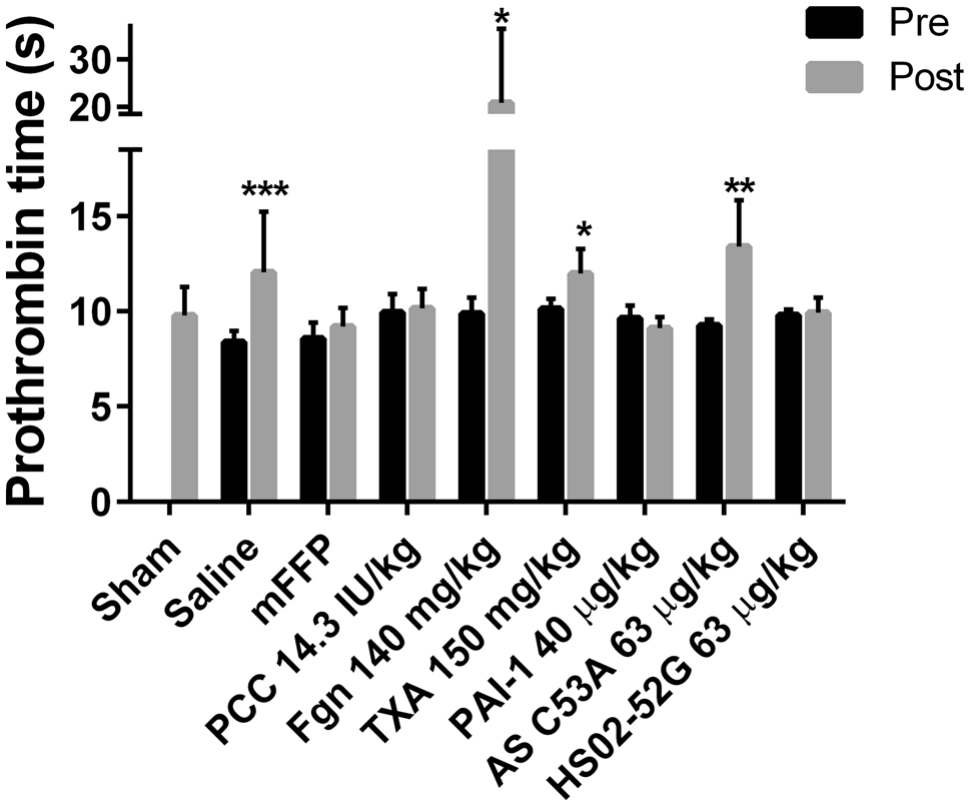
Clotting times pre- and post-treatment. Prothrombin time (PT) values (s) are shown for mice whose blood loss values are shown in Fig. 3. Pre (black bars), from plasma of blood sampled prior to haemorrhagic shock (HS) and resuscitation with fluids and doses shown on x axes and liver laceration (LL); Post (grey bars), from plasma of blood sampled after HS/LL and resuscitation. Values are the mean of 6 determinations ± SD in all cases except n = 8 for saline. Asterisks above “Post” bars indicate statistically significant differences from “Pre” values by Mann–Whitney U tests (*p < 0.05, **p < 0.01, ***p < 0.001).

### Biomarkers of coagulation and fibrinolysis

Plasma samples were tested for coagulation and fibrinolysis biomarkers (aPC, tissue plasminogen activator (tPA), plasminogen activator-1 (PAI-1), and D-dimer) (Fig. 5). Functional aPC levels in plasma samples from sham-treated mice were 900 ± 300 pg/ml, which did not differ from all other pre-HS/LL values (Fig. 5a). In contrast, saline-treated mice exhibited a statistically significant 8.5-fold increase in aPC activity (pre-HS/LL 900 ± 200 pg/ml vs. post-HS/LL 8000 ± 2000 pg/ml, *p* < 0.01). This elevation was eliminated in mice treated with HS02-52G or PAI-1 and reproduced in mice treated with control aptamer AS C53A (8.2-fold post-HS/LL vs pre-HS/LL increase). Treatment with mFFP also suppressed a statistically significant increase in aPC levels. PCC reduced but did not eliminate post-HS/LL elevation, in that aPC levels were significantly elevated 3.8-fold post-HS/LL versus pre-HS/LL but did not differ significantly from sham.

**Figure 5:**
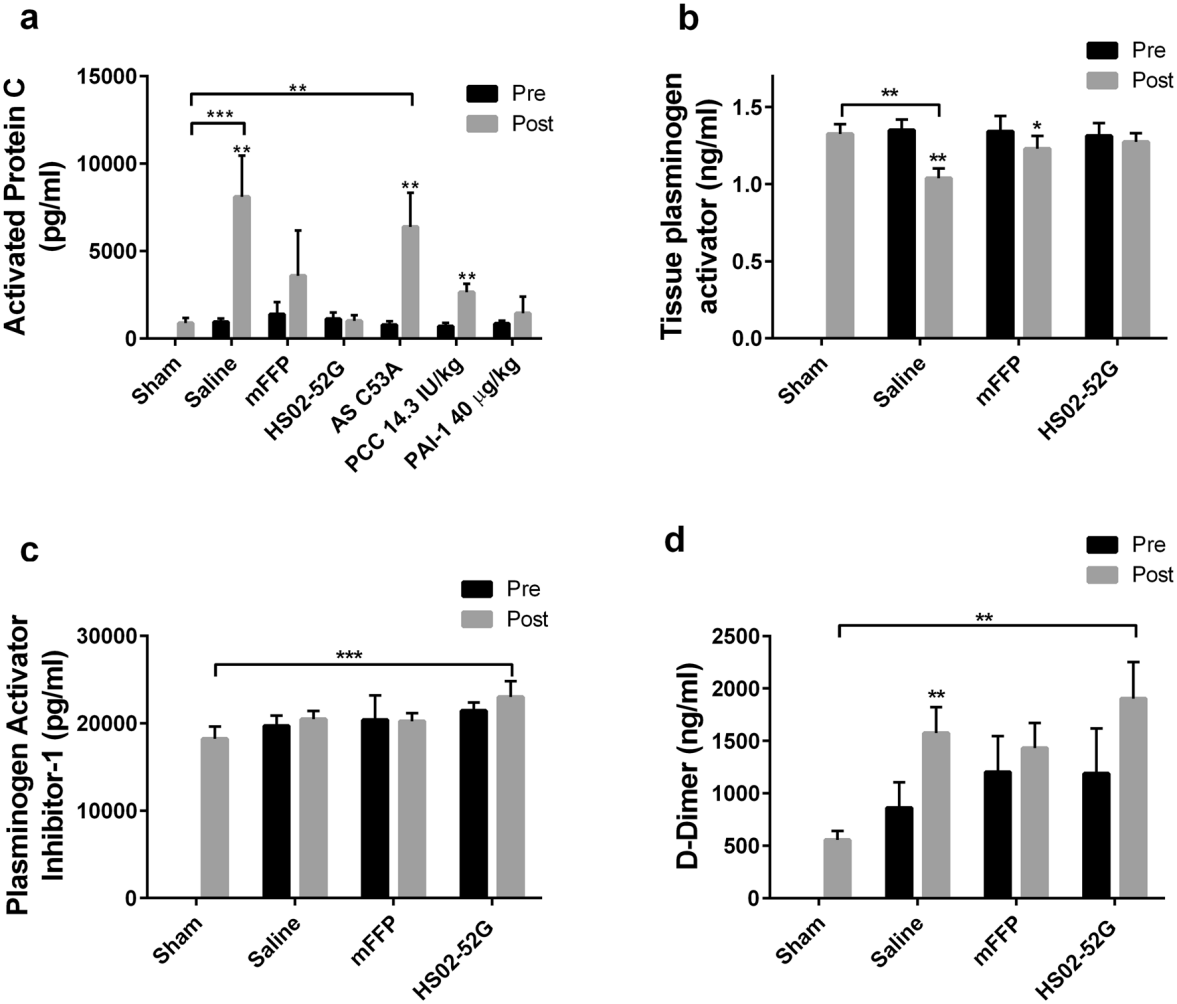
Coagulation and fibrinolytic markers in plasma from HS model mice. Plasma samples taken Pre-HS and Post-HS/resuscitation/liver laceration were assayed for the activities of activated protein C (**A**), tissue plasminogen activator (**B**), and plasminogen activator inhibitor-1 (**C**), as well as the concentration of D-dimer (**D**). Pre and Post, as in Figs. 3 and 4. The means of 6 determinations ± SD are shown in (**A**) and 5 determinations ± SD in (**B**), (**C**), and (**D**). Asterisks atop “Post” bars indicate statistical differences from corresponding Pre values by Wilcoxon test; asterisks above horizontal brackets refer to statistical differences between groups Kruskal–Wallis test with Dunn’s post-tests. In both instances: *p < 0.05, **p < 0.01, ***p < 0.001.

Less notable changes were observed with three biomarkers related to fibrinolysis: tPA, PAI-1, and D-dimer. Saline treatment lead to significantly lower levels of tPA post-HS/LL versus pre-HS/LL or versus sham-treated mice, a decline of 24–28%, respectively (Fig. 5b). A lesser, but still significant reduction was also observed in mice treated with mFFP, while no significant change was noted in mice treated with HS02-52G. PAI-1 activity levels did not differ significantly pre-HS/LL versus post-HS/LL for saline-, mFFP-, or HS02-52G-treated mouse plasmas; among the three post-HS/LL conditions, only HS02-52G treatment was associated with a significant elevation (Fig. 5c). D-dimer, a fibrin degradation product indicative of activation of both coagulation and fibrinolysis^25^, was significantly elevated post-HS/LL in saline-treated mice, but no significant post- versus pre-treatment change was seen with either mFFP, and HS02-52G, although post-treatment HS02-52G-treated values were significantly elevated over sham.

The profile of minimal effects on fibrinolysis noted above was reinforced by the results of in vitro fibrinolysis assays in which plasma samples from HS/LL mice were recalcified and exposed to tissue factor, followed by exogenous tPA, and turbidity was monitored over time. Mice treated with mFFP, Fg, or PAI-1 exhibited minimal but detectable in vitro fibrinolysis unchanged between pre- and post-HS/LL samples. TXA-treated mice showed significantly decreased in vitro fibrinolysis post-HS/LL, as expected. Pre-HS/LL samples from saline-treated or PCC-treated mice showed minimal but detectable fibrinolysis that was overridden by renewed fibrin formation in post-HS/LL samples (see Supplementary Fig. S3), a statistically significant change, although in no case did the mean change in milliOD_405_/minute values exceed ± 0.001.

### Endothelial glycocalyx biomarker

Syndecan-1 (sdc1) is an endothelial glycocalyx marker; soluble sdc-1 is shed by stressed endothelial cells, although it is not clear if such shedding indicates endothelial damage^26^. Levels of sdc-1 were significantly increased in all post-HS/LL groups relative to pre-HS/LL values, on average by 2.9- to 6.3-fold, when saline, mFFP, PA1-I, and high-dose fibrinogen and TXA treatments were compared, although only post-HS/LL TXA-treated levels significantly exceeded those observed in sham-treated mice (Supplementary Fig. S4). Table 1 summarizes the results of all treatments with respect to blood loss, clotting times, aPC, tPA, PA1-1, D-dimer, and sdc-1.

**Table 1:** Summary of findings. For principal outcomes (blood loss and coagulopathy) “✓” signifies positive finding and “X” signifies negative finding; “?” signifies indeterminate finding, with high variance and possible interference from pre-existing aggregates in some samples.

Treatment	Normalized blood loss	Eliminated coagulopathy (PT)	aPC activity (Pre- vs. Post)	tPA activity (Pre- vs. Post)	PAI-1 activity (Pre- vs. Post)	D-dimer levels (Pre- vs. Post)	Syndecan-1 levels (Pre- vs. Post)
Saline	X	X	↑↑↑	↓	–	↑	↑↑↑
mFFP	✓	✓	–	–	–	–	↑↑↑
PCC 14.3 IU/kg	✓	✓	↑	ND	ND	ND	ND
Fibrinogen 140 mg/kg	✓	?	ND	ND	ND	ND	↑↑
PAI-1 40 µg/kg	X	✓	–	ND	ND	ND	↑↑
TXA 150 mg/kg	✓	X	ND	ND	ND	ND	↑↑↑
AS C53A	X	X	↑↑↑	ND	ND	ND	ND
HS02-52G	✓	✓	–	–	=	↑	ND

For additional outcomes (aPC, tPA, PAI-1, D-dimer, and syndecan-1), ↑↑↑ indicates major increase, ↑↑ indicates intermediate increase, ↑ indicates increase, ↓ indicates decrease, – indicates no change, and ND indicates not determined, all Post-HS/LL values relative to Pre-HS.

### Cytokines

Cytokine arrays were employed to survey a panel of 40 cytokines or growth factors for changes associated with HS and fluid resuscitation without liver laceration before and after treatment with mFFP, 14.3 IU/kg PCC, and 140 mg/kg Fg. Normal murine pooled plasma (NMP) was also probed. Results were expressed as ratios (pre-HS/NMP, and post-HS/pre-HS for the three treatments) (Supplementary Table 1). All factors exhibited higher levels in pre-HS samples, which had undergone tissue trauma, than in NMP samples obtained via cardiac puncture, with the five factors most elevated being: sICAM-1, IL-13, KC, MIP-1β, and RANTES. With respect to post-/pre-treatment ratios, five different factors exhibited strong elevation (ratios > 11) for all three resuscitation fluids: IL-6; IL-10; KC; JE; MIP-2; and TNF-α.

### Plasma dose effects

The dose of mFFP was reduced to match a clinically recommended human plasma dose (12 ml/kg)^27^. This reduction was achieved within the HS/LL model by keeping the total resuscitation volume equal to the volume of blood withdrawn for shock, and making up the difference with saline (e.g. if 0.7 ml of blood was drawn from a 25 g mouse to reduce MAP to 35 mm Hg, then 0.3 ml of mFFP and 0.4 ml of saline were administered for a total of 0.7 ml resuscitation fluid). Mice receiving 12 ml/kg mFFP lost 170 ± 30 mg of blood, still significantly less than saline-treated animals (240 ± 30 mg, p < 0.01) (Supplementary Fig. S5a) but more than mice receiving mFFP to replace all shed blood volume (140 ± 30 mg) (Fig. 3). In contrast to full-dose mFFP mice, 12 ml/kg mFFP-treated mice were coagulopathic after HS/LL; PT values were significantly elevated, on average by 35% as evidenced by statistically significant increased clotting times for PT (Supplementary Fig. S5b). Mice treated with 12 ml/kg mFFP exhibited significantly attenuated, 5.8-fold less aPC elevation compared to saline-treated animals (post-HS/LL aPC levels of 1400 ± 500 ng/ml), although post-HS/LL levels remained significantly higher than pre-HS/LL levels (Supplementary Fig. S5C).

### Absence of antitrypsin in PCC

HS02-52G inhibits aPC^24^ and mFFP contains α_1_-antitrypsin (AAT), a natural inhibitor of APC^28^. PCC is enriched in vitamin K-dependent proteins but contains other plasma proteins^29^. Immunoblotting with an anti-AAT antibody was used to assess if AAT was present in PCC. Supplementary Fig. S6 shows a duplicate polyacrylamide gel and immunoblot probed with anti-AAT antibodies. Under conditions in which at least 25 ng of purified AAT was detected and 1000 ng of an unrelated protein (human albumin) was not, no AAT band was detected in up to 5000 ng of PCC. This finding suggests that if AAT is present in PCC, it is at levels of 1% or less, on a weight basis.

## Discussion

Our principal study outcomes were blood loss and prothrombin time amelioration after fluid resuscitation and haemostatic challenge. With respect to blood loss, we found that measures that supported coagulation, either directly or indirectly, restored haemostasis and eliminated the bleeding diathesis induced by trauma and haemorrhagic shock in HS/LL mice. These included mFFP, HS02-52G, fibrinogen, PCC, and TXA. Crystalloid (saline) or colloid (5% HAS) were ineffective anti-haemorrhagic treatments, showing that amelioration of shock alone was insufficient to restore haemostasis, as was PAI-1 infusion. Plasma transfusion and fibrinogen and PCC concentrate infusion are directly procoagulant via provision of clotting factors. HS02-52G is indirectly procoagulant via inhibition of anticoagulant aPC^24^. TXA is an anti-fibrinolytic agent, a lysine analogue that could promote coagulation via inhibition of fibrinolysis^30^, and whose use has been shown to reduce mortality in trauma-haemorrhage patients in the CRASH-2 trial^31^. Although we administered PAI-1 at a supraphysiological dose, its observed anti-haemorrhagic inferiority versus TXA in HS/LL may have arisen either from the ability to give higher molar doses of TXA and/or from differing mechanisms of action of these two anti-fibrinolytic agents^30^.

Our other principal outcome was elimination of coagulopathy. Because trauma patients with the worst prognosis are both bleeding and coagulopathic, we focused on interventions in our model that reduced bleeding to sham or pre-HS levels and which were associated with no change in PT after HS/LL versus pre-HS values. Only three treatments met both criteria: mFFP; PCC; and HS02-52G. While it could be argued that fibrinogen treatment met both criteria, at the dose that was anti-haemorrhagic, post-HS/LL PT results in some mice in that cohort were obscured by the presence of pre-formed aggregates in plasma that could constitute a prothrombotic safety signal. High doses of Fg concentrates are required to raise fibrinogen levels in trauma-haemorrhage patients^32^. TXA eliminated the ATC/TIC-associated bleeding diathesis in HS/LL mice at the dose employed,based on rabbit models^33,34^, but did not eliminate PT-defined coagulopathy. This finding is perhaps unsurprising given that the PT uses high concentrations of tissue factor that promote clotting within 10–14 s and has little or no capacity to assess clot lysis^35^.

Like other investigators using rodent models of ATC/TIC^36–38^, we observed a substantial elevation in plasma aPC activity after trauma and haemorrhagic shock. Saline-treated mice exhibited the greatest increase in aPC levels of groups surveyed, an 8.5-fold elevation indicative of an active process, given its magnitude and opposite direction from any dilutional effects. The elevation in aPC activity was eliminated by anti-aPC aptamer treatment with HS02-52G and correlated with ATC/TIC reversal, in that blood losses returned to sham levels on haemostatic challenge and coagulopathy was eliminated; plasma (mFFP) transfusion had similar effects. Inhibition of aPC by plasma transfusion is possible because plasma contains high levels (~ 40 µM) of the serine protease inhibitor AAT, which is a physiological inhibitor of aPC^28^ and murine plasma contains twice as much AAT as human plasma^39^. However, aPC inhibition cannot explain the effectiveness of PCC treatment on both bleeding and coagulopathy parameters. PCC is a plasma protein product and factor concentrate enriched in coagulation factors II, VII, IX and X and proteins S and C^40^. It is known to contain other plasma proteins, but AAT has not been reported to be one of them, and we could not detect AAT in the PCC preparation we employed^29^. PCC therefore likely exerts a procoagulant effect by increasing the concentrations of the procoagulant factors listed above, facilitating their activation by mass action. We previously found PCC to reduce bleeding and eliminate coagulopathy in a normovolemic mouse model of pan-plasma factor depletion achieved by sequential blood exchange^23^.

In this study, we adapted the previously described mouse ATC/TIC model of Chesebro et al., comprising anaesthesia, trauma, and pressure-defined haemorrhage^36^. These previous investigators performed a 2-cm mid-line laparotomy to model soft tissue trauma, and they cannulated femoral vessels for pressure measurement and blood sampling, closing incisions prior to allowing their C57BL/6 J mice to emerge from anaesthesia. Their pressure-defined haemorrhage to 35 ± 5 mM Hg and 60 min of shock, was performed on conscious mice restrained on a board. We adapted this model to include fluid resuscitation and subsequent haemostatic challenge, to match available instrumentation, and to address local animal research ethics requirements. Our HS/LL model was performed under full anaesthetic cover at all times, in CD1 mice, with soft tissue trauma modelled by excising a 1 × 1 cm neck skin and tissue flap and cannulating the exposed carotid artery, and by incising the leg and inserting a pressure probe into the exposed femoral artery. We employed the same shock target pressure and shock period as Chesebro et al., resuscitated with fluids equal to the shed blood volume, and assessed haemostasis via the bleeding challenge of liver laceration. Chesebro et al. found that only the combination of trauma and haemorrhagic shock led to coagulopathy, as judged by activated Partial Thromboplastin Time (aPTT) elevation. We found that trauma alone induced coagulopathy, as judged by elevated PT values in sham-treated mice (no haemorrhagic shock, no resuscitation) versus control (no additional manipulations save cardiac puncture). Because of our focus on resuscitation, we tested for coagulopathy after haemorrhage, but before the shock period, and after resuscitation and haemostatic challenge. We cannot, therefore, exclude the possibility that the TIC that we observed intensified after hemorrhagic shock and abated partially after resuscitation. Some resuscitation fluids, like saline, further exacerbated TIC in HS/LL, suggesting an additional dilutional coagulopathic effect, while some, like plasma, did not. HS/LL is therefore a model of ATC/TIC, which differs from the Chesebro et al. model in mouse strain, anaesthesia, the nature of soft tissue trauma, and in the use of fluid resuscitation and subsequent hemostatic challenge. It is pragmatically focused on our primary research question of which resuscitation fluids restored haemostatic control and ameliorated coagulopathy, and which did not. Others have also modified the original Chesebro et al. model to include full anaesthetic coverage in mice and rats^37,38,41^.

Reducing MAP to 35 mM Hg required the removal of ~ 0.7 ml of blood in the HS/LL model. For the 30 g mice we typically employed, at an estimated blood volume of 6–8% of body weight, or 1.8–2.4 ml, this comprised a substantial blood loss of 29–39% blood volume, which corresponds to the most severe shock class III or IV in patients^42^. Since pressure-defined haemorrhage involves blood removal via a cannula, and not via an injury, to assess haemostasis it was necessary to create a bleeding injury. Because of shock-related peripheral vasoconstriction, we could not assess haemostasis via tail transection, finding in initial studies that HS/LL resuscitated mice bled very little from this peripheral injury. We therefore selected standardized liver laceration as the haemostatic challenge, based on our previous work in mice rendered coagulopathic via anticoagulant administration^43^ or blood exchange^22,23^.

Hyperfibrinolysis has been reported in clinical ATC/TIC, using either standard tests (e.g. D-dimer) or viscoelastometric assessment of in vitro clot lysis^14^. In a pressure-defined HS model, a three-fold elevation of D-dimer was reported post- versus pre-HS in wild-type mice, an elevation not seen in transgenic mice with reduced capacity to activate protein C^41^. We observed a 1.8-fold significant elevation of D-dimer post-HS/LL versus pre-HS, but significant differences were eliminated by mFFP or HS02-52G treatment. Given that saline treatment slightly suppressed tPA levels, an antifibrinolytic suppression attenuated by HS02-52G but not mFFP treatment, and that post-HS/LL plasmas from saline-treated mice failed to lyse in vitro, it seems unlikely that suppression of hyperfibrinolysis was mechanistically linked to the suppression of ATC/TIC observed for some treatments in our model. D-dimer elevation could simply indicate an elevation in both clot formation and clot lysis, one expected given the degree of haemostatic challenge in our model.

ATC has also been associated with changes in other biomarkers indicating inflammation or endothelial damage^14^. Although our cytokine array experiments were less extensive than a previous analysis of cytokine profiles in a rat model of polytrauma and 40% haemorrhage^44^, the results were partially concordant in that elevations in KC, RANTES, IL-6, IL-10, and TNF-α were noted in both studies. Similarly, we examined one marker of endothelial glycocalyx damage, soluble sdc-1. This marker was elevated post-HS/LL versus pre-HS/LL both for treatments alleviating ATC (e.g. mFFP) and for ineffective treatments (e.g. saline, PAI-1). Similar findings for this marker were observed in a rat pressure- and lab-guided HS model^45^, supporting the deduction that while some form of endothelial damage or activation is a component of ATC, it does not necessarily need to be normalized to achieve haemostasis. We might also speculate that while a cytokine storm does seem to be found in ATC/TIC models, including HS/LL, it need not be reversed to achieve haemostasis.

There are several limitations to this study related to choices made in the model and in the nature of the interventions tested. Firstly, the combination of resuscitation and treatment fluid into one step simplified animal handling but magnified differences from clinical trauma and current recommendations for low volume prehospital fluid resuscitation^42^. Using undiluted mFFP as the resuscitation fluid led to administration at a dose of 28 ml/kg, substantially higher than clinical dose recommendations of 10–15 ml/kg^27^. Reducing the mFFP dose to 12 ml/kg was still associated with a significant reduction in blood loss, but it was no longer effective in eliminating coagulopathy, possibly because we supplemented the mFFP with saline to keep the volume of resuscitation fluid high and constant across all interventions. Secondly, it is conceivable that a higher dose of interventions may be superior to those we tested, although weight-based doses aligned to clinical treatments were employed wherever possible. Thirdly, for PCC and Fg we employed human plasma protein products in mice because no equivalent murine concentrates were either commercially available or practical to manufacture ourselves. We and others have used these human protein concentrates in mice, and while murine proteins could be more physiologically compatible, the high degree of homology between murine and human coagulation factors mitigates this concern to some degree. Fourthly, while others have followed blood gas, lactate, hemoglobin and platelet levels and activities in similar rodent models of TIC^36,37^, such measurements were beyond the scope of the current study. Fifthly, we did not assess the safety of our interventions, in that it is possible that some procoagulant or antifibrinolytic treatments could have caused localized thrombosis. Pre-formed aggregates in plasma from some mice in our high-dose fibrinogen cohort may be indicative of such a problem. Sixthly, the potential for Type 1 errors is particularly acute in a study testing multiple interventions such as this one, one that can only be partially addressed through the use of multiple comparisons statistical testing methods; future studies can address the false discovery possibility by focusing on our most robust positive findings.

In future, we plan to investigate platelet parameters, refine our understanding of the temporal emergence of coagulopathy, and vary the volume of resuscitation fluid to understand if smaller volume interventions with higher translational potential are effective in ameliorating the bleeding diathesis and coagulopathy in our model. Further investigations of aPC inhibition via HS02-52G are also warranted to determine optimal doses and if prophylactic administration is effective. Prophylactic administration of a variant form of recombinant FVa resistant to aPC inactivation (^Super^FVa) has been reported to reduce blood losses significantly in mice subjected to extensive hepatectomy leading to uncontrolled haemorrhage^38^.

## Conclusions

Multiple treatments resolved both the bleeding diathesis and the coagulopathy associated with ATC/TIC in a mouse model of hemorrhagic shock. Their common feature was the direct or indirect promotion of coagulation. This profile was most clearly demonstrated by HS02-52G, a DNA aptamer and specific inhibitor of aPC not previously employed in vivo. Further investigations of this agent and of other aPC inhibitors in ATC/TIC are warranted.

## Materials and methods

### Mouse model of haemorrhagic shock with liver laceration (HS/LL)

CD-1 mice (25–30 g, Charles River, St-Constant, QC, Canada) were employed for in vivo experiments conducted with approval from the Animal Research Ethics Board of the Faculty of Health Sciences of McMaster University, using equal numbers of male and female in all groups (Animal Utilization Protocols 16-04-13 and 2020-02-06 to WPS). All experimental methods involving animals were performed in accordance with these protocols and all relevant regulations and guidelines. Mice always had unrestricted access to regular chow and water and were housed under a consistent cycle of 12-h light and 12-h dark. This study was performed in accordance with ARRIVE guidelines (https://arriveguidelines.org)^46^.

The HS/LL model combines elements of traumatic injury and haemorrhagic shock with fluid resuscitation and haemostatic challenge. Figure 1b shows a timeline of specific actions in the HS/LL model. Mice were anesthetized via intraperitoneal injection of 2.5% (w/vol) tribromoethanol (Sigma-Aldrich, Oakville, ON, Canada) and maintained on a heating pad. The femoral artery was surgically exposed, and a 1F Millar high fidelity rodent pressure catheter was inserted and connected to a Transonic PowerLab system (Transonic Systems, Ithaca, NY, USA) to monitor Mean Arterial Pressure (MAP). A 1 × 1 cm skin flap was excised from the neck and incisions exposed the carotid artery, which was cannulated using PE10 tubing. The neck area excision and the leg incision together comprised the tissue trauma in HS/LL. Ten minutes after catheterization, blood was removed via the cannula, over a five-minute period, until the MAP reached 35 mM Hg, and anticoagulated with 1: 9 parts 3.8% (w/vol) sodium citrate. MAP was maintained at 35 ± 5 mM Hg for 60 min. Shed blood volume was measured and an equal volume of resuscitation fluid was administered slowly via the cannula over a 2- to 3-min period. Pre-procedure plasma from shed blood was obtained via microcentrifugation at room temperature in an AccuSpin Micro 17R Centrifuge (Fisher Scientific, Mississauga, ON, Canada) 5 min at 17,000 g) and immediately frozen at − 80 °C as previously described. Over the next five minutes, the abdominal cavity was opened, and a liver lobe was exposed and exteriorized. A 5 mm perforating transverse incision was made through the lobe’s inferior edge using a #10 scalpel blade. Shed blood was captured into a tared plastic weigh boat for 15 min post-liver laceration and combined with any blood clotted on the surface of the injured lobe. Post-procedure citrated plasma was processed from a terminal cardiac puncture blood sample as described above, and frozen at -80 °C for subsequent analysis. Mice were euthanized by exsanguination and cervical dislocation. In some experiments control blood samples were obtained from anaesthetized mice by cardiac puncture or from anesthetized sham mice subjected to tissue trauma but not shock.

A total of 150 mice were entered into this study. Results from all mice surviving to the study endpoint were included. Results were excluded from 5 mice who died unexpectedly before reaching the study endpoint, and results were replaced with those of additional mice meeting the inclusion criteria, as planned a priori (see Supplementary Data for additional information). Results from 150 mice were therefore employed for analysis. Randomization was not employed. The order of treatments and measurements and of cage locations was varied to avoid confounding. Blinding was not employed. The principal outcomes of the study were the amount of blood lost after liver laceration and before and after PT values; other outcomes were in vitro plasma parameters measured to explore potential mechanisms.

### Resuscitation fluids

Mice were resuscitated with: 0.9% (w/vol) normal saline; murine fresh-frozen plasma (mFFP); prothrombin complex concentrate (PCC, Octaplex, Octapharma, Toronto, ON, Canada), human fibrinogen (free of von Willebrand factor, plasminogen, and fibronectin, Enzyme Research Laboratories [ERL], South Bend, IN, USA); tranexamic acid (Sigma-Aldrich); mouse Plasminogen Activator Inhibitor-1 (PAI-1, Innovative Research, Novi, MI, USA); or oligonucleotides. Oligonucleotides synthesized by Integrated DNA Technologies, Inc. (Coralville, IA, USA) were: 52 base anti-aPC aptamer HS02-52G^24^ (5′-GCCTCCTAAC TGAGCTGTAC TCGACTTATC CCGGATGGGG CTCTTAGGAG GC-3′); and 51 base control oligonucleotide AS C53A (5′- AGTGAATTCT TAGTGATGGT GATGGTGATG AATGGCGCTG CCTGCCACGG C-3′). Saline was used to dilute any products requiring dilution prior to administration.

### Clotting assays

Clotting assays employed a haemostasis analyser (Start4, Diagnostica Stago, Asnières, France). Plasma samples stored at − 80 °C were thawed in a 37 °C water bath prior to testing. Prothrombin time (PT) was determined by combining 50 µl of citrated plasma with 50 µl of Thromborel S PT reagent (Siemens, Marburg, Germany). To assay the aPC-mediated prolongation of the aPTT, 22.5 µl of normal human pooled plasma was combined with 2.5 µl of various concentrations of HS02-52G or AS C53A in saline and 25 µl of 0.2 µg/ml activated Protein C (aPC, ERL) and incubated for 2 min at 37 °C, prior to addition of 50 µl PTT-A aPTT reagent, and, 3 min later, 50 µl of 25 mM CaCl_2_ to initiate clotting.

### Biomarker assays

Functional activity biomarkers in mouse citrated plasma samples were measured using Enzyme-Linked Immunosorbent Assay (ELISA) kits (96 well microtiter plate format): aPC (Mouse Activated Protein C (APC) ELISA Kit, (Abbexa Ltd., Cambridge, United Kingdom); tissue Plasminogen Activator, tPA (Mouse PLAT/TPA ELISA Kit, LSBio, Seattle, WA, USA); and Plasminogen Activator Inhibitor-1, PAI-1 (Invitrogen™ PAI-1 (SERPINE1) Mouse ELISA Kit, Invitrogen/Thermo Fisher Scientific, Ottawa, ON, Canada). Other kits measured total plasma levels of: D-dimer (Mouse Fibrin Degradation Product D-Dimer (Competitive EIA) ELISA Kit, LSBio); and syndecan-1 (Mouse Syndecan-1 ELISA kit, Abcam, Waltham, MA, USA).

### Plasma clot lysis assay

Mouse plasma was assayed in a 96-well plate (final concentration 35%) in HEPES buffered saline containing 0.1% PEG. Clotting was initiated by the addition of a 1/30,000 dilution of Innovin (Siemens) and 16 mM CaCl_2_ with penicillin/streptomycin (total volume 100 µl). Plasma was clotted for 120 min at 37 °C and turbidity at 405 nm was measured every 5 min on a plate reader. 20 µl of 300 nM Tenecteplase (Genentech/Roche, South San Francisco, CA, USA) in HEPES buffered saline containing 0.1% PEG and 0.01% Tween 20 was layered on the clots and readings resumed every 5 min for 180 min. Results were plotted in Microsoft Excel and maximum rate of clot lysis was calculated by deriving the slope.

### Mouse cytokine array

Proteome Profiler Mouse Cytokine Array Kits, Panel A (R&D Biosystems/Bio-Techne Canada, Toronto, ON, Canada) were probed using 200 µl citrated mouse plasma per array and two arrays per treatment. Arrays were imaged using an Azure Biosystems 500Q gel documentation system (exposure time, 10 min, pixel binning set to 1 × 1). Spots were quantified using AzureSpot (Azure Biosystems, Dublin, CA, USA) software with spot E9 set as background. Results are reported as ratios of the average pixel density units between duplicate blots for post-procedure/pre-procedure comparisons.

### Sample sizes

Sample sizes were determined via power calculations (GraphPad Statmate 2.0, GraphPad Software, San Diego, CA, USA), using pilot data from our previous model of murine coagulopathy and liver laceration^23^, which revealed that with α at 0.05, SD (σ) of 33.5 mg, n = 6 was predicted to give 80% power to detect a significant difference in means of 65 mg of blood lost.

### Data presentation and statistical analysis

Data are presented as the mean ± one standard deviation (mean ± SD) in either bar graph or scatter plot formats. Graphs were generated using GraphPad Prism version 6.07 (GraphPad Software) and statistical analysis was carried out using the same software, with significance declared at *p* < 0.05. Only non-parametric tests of significance were used in this study. Comparisons of three or more data sets used Kruskal–Wallis tests with Dunn’s post-test for multiple comparisons; comparisons of two data sets employed Mann–Whitney U tests.

## Supplementary Information

Supplementary Information.

## Data Availability

All datasets generated during and/or analysed during the current study are available from the corresponding author on reasonable request.
