# Supplementary material for: Correction of haemorrhagic shock-associated coagulopathy and impaired haemostasis by plasma, prothrombin complex concentrates or an activated protein C-targeted DNA aptamer in mice
Source: Sci Rep. 2023 Mar 7;13:3811. doi: 10.1038/s41598-023-30794-7 (PMC9992365; doi:10.1038/s41598-023-30794-7)
Supplement: Supplementary file 1 — Supplementary Information. [file 41598_2023_30794_MOESM1_ESM.pdf]

Supplementary Data for:

Correction of haemorrhagic shock-associated coagulopathy and impaired haemostasis by plasma, prothrombin complex concentrates or an activated protein C-targeted DNA aptamer in mice

Louise J. Eltringham-Smith[1], Scott C. Meixner[2,3], Edward L. G. Pryzdial[2,3], and William P. Sheffield[1,4,*].

Medical Affairs and Innovation, Canadian Blood Services, Hamilton, ON[1], and Vancouver BC[2], Canada,
[3]Centre for Blood Research, University of British Columbia, Vancouver, BC, Canada, and
[4]Department of Pathology and Molecular Medicine, McMaster University, Hamilton, ON, Canada.

Short running head: Coagulopathy and haemostasis in murine haemorrhagic shock

Supplementary Information

Materials and Methods

Mouse model of haemorrhagic shock with liver laceration (HS/LL). As noted in the text of the main manuscript, 5 of 150 mice died after haemorrhagic shock. Two of these mice were planned to be resuscitated with mFFP and one each with 5% Human Serum Albumin, one with 140 mg/kg fibrinogen, and one with 14.3 IU/kg Prothrombin Complex Concentrate. These five mice were replaced with mice treated with the same agents as listed above but who survived until the study endpoint and were therefore eligible for inclusion.

Supplementary Figures and Table

Figures

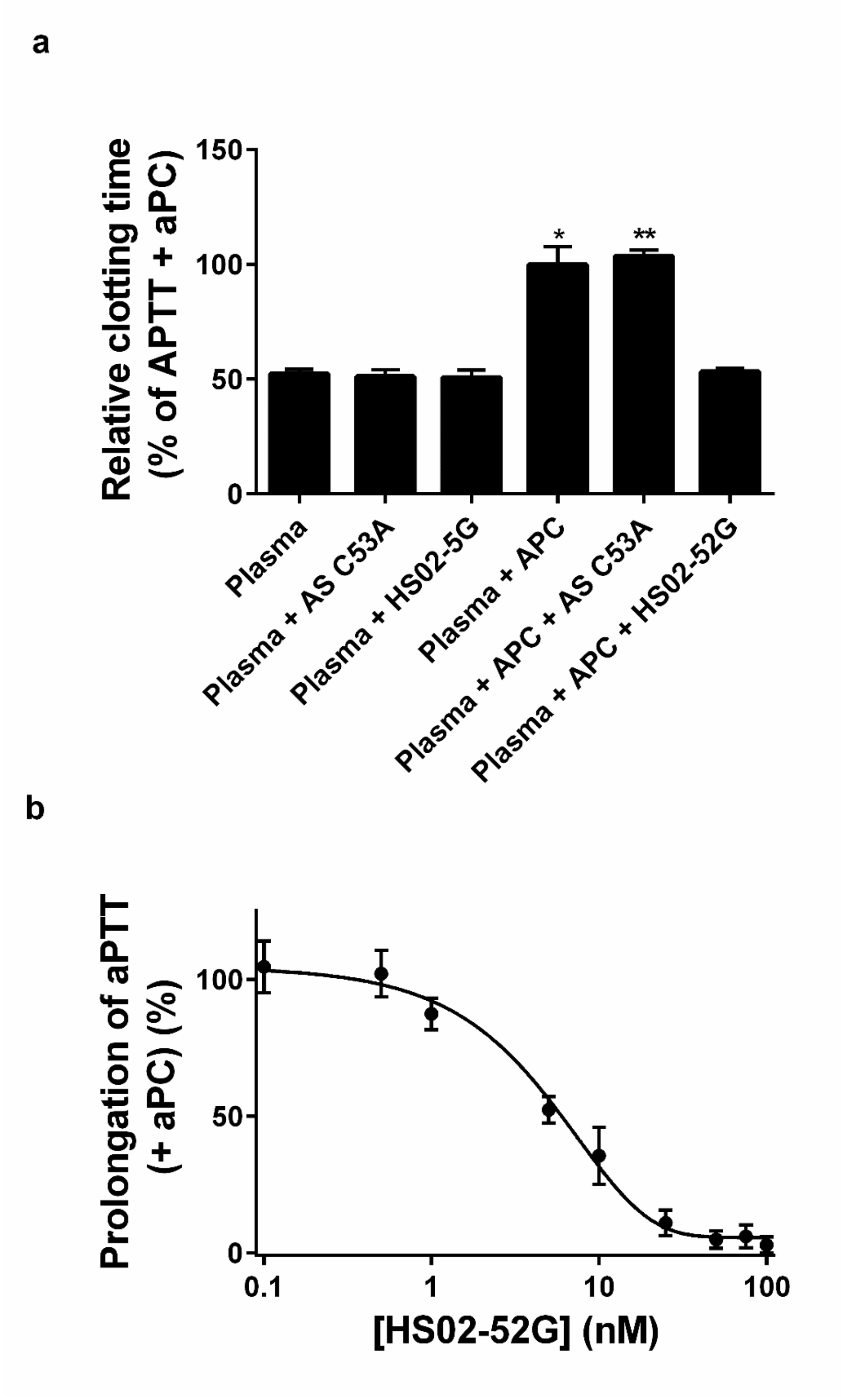

Supplementary Figure 1. Relative clotting times for modified Activated Partial Thromboplastin Time (APTT) supplemented with activated Protein C (aPC). Panel **a**, normal human pooled plasma was pre-incubated with or without 0.2 mg/ml aPC, combined with aPTT reagent, recalcified, and clotting time determined in the presence or absence of control

oligonucleotide AS C53A or anti-APC DNA aptamer HS02-52G (100 nM final concentration). The mean of 8 determinations ± SD is shown. Asterisks above the bar indicate statistically significant difference from plasma only control by Kruskal-Wallis test with Dunn's post-tests ($p < 0.05$, *, $p < 0.01$, **). Panel **b**, as in **a**, but the concentration of HS02-52G was varied from 0.1 to 100 nM.

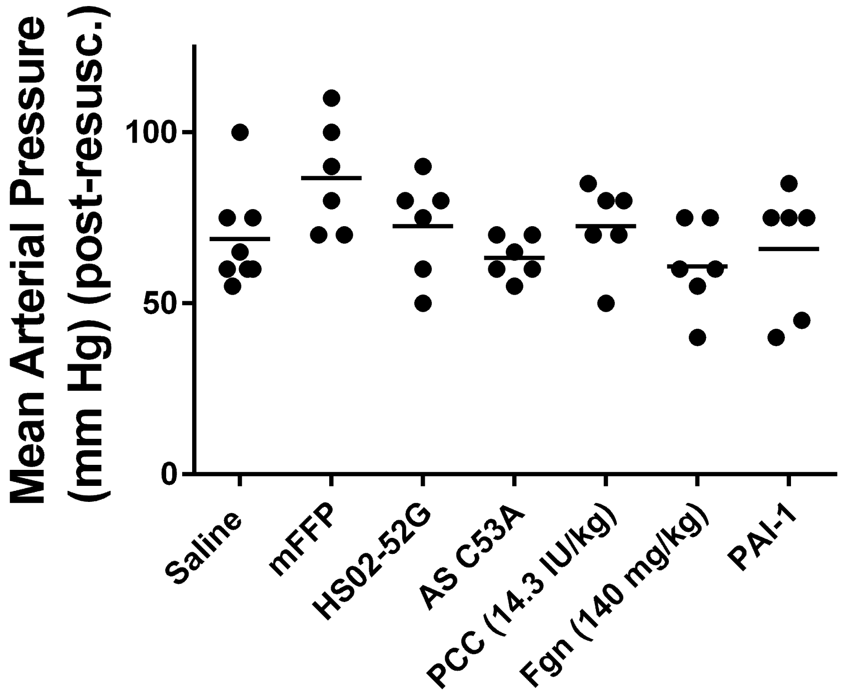

Supplementary Figure 2. Mean Arterial Pressure (MAP), in mM Hg, is shown for the same groups of mice whose blood losses are shown in Fig. 3, identified by resuscitation fluid on the x axis. Post-resuscitation (post-HS/LL) values are shown. The mean (horizontal line) 6 determinations is shown for all groups, except for Saline for which 8 determinations are shown. None of the differences were statistically significant by Kruskal-Wallis test.

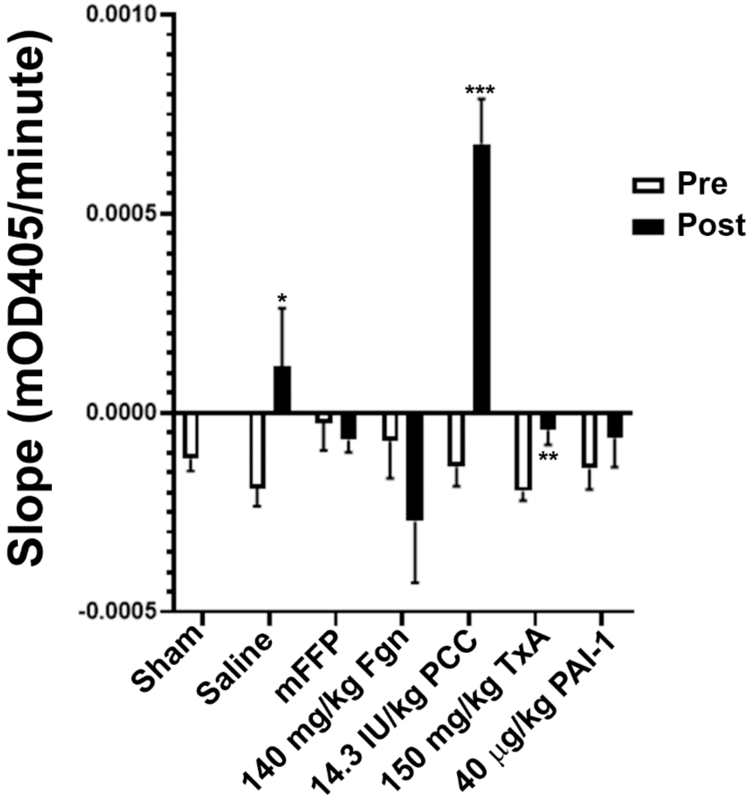

Supplementary Figure 3. Rate of in vitro fibrinolysis. The HS model protocol (Fig. 1) was modified to substitute liver laceration for a rest period after resuscitation. Pre- and post-procedure blood sampling was used to obtain plasma samples (white and black bars, respectively) for in vitro fibrinolysis following resuscitation with treatment fluids identified below the x axis. Samples were recalcified and clotted with dilute commercial tissue factor, then tPA (Tenecteplase) was layered on the clot and the optical density at 405 nm (OD405) was followed in a plate reader. The mean rate of change of the optical density (mOD405/min) was then determined and is shown for Pre (white) and Post (black) samples (n=6 ± SEM). Negative values indicate fibrinolysis and positive values indicate clot growth. * denotes $p < 0.05$, ** $p < 0.01$, and *** $p < 0.001$ versus Pre value by Mann-Whitney U test.

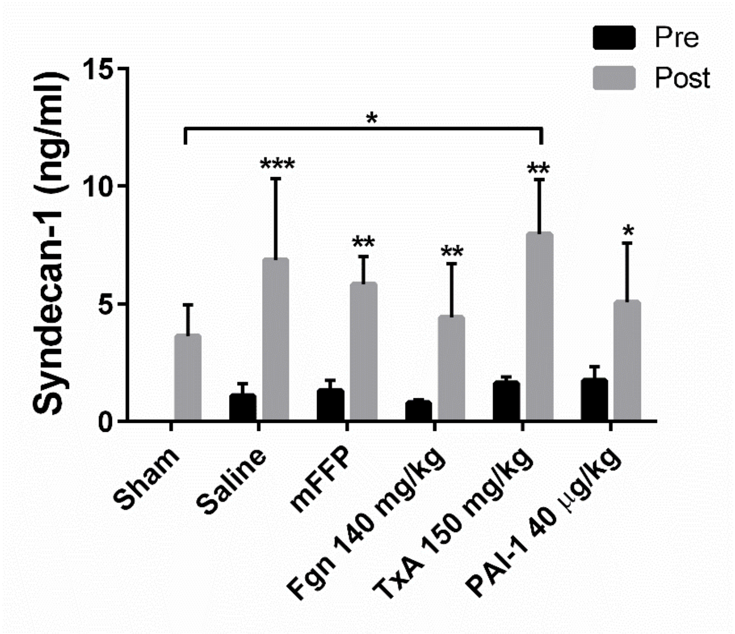

Supplementary Figure 4. Soluble syndecan-1 in plasma from HS model mice. Plasma samples taken Pre-HS and Post-HS/resuscitation/liver laceration were assayed for soluble syndecan-1 (ng/ml). Pre (black bars), from plasma of blood sampled prior to haemorrhagic shock (HS) and resuscitation with fluids and doses shown on x axes and liver laceration (LL); Post (grey bars), from plasma of blood sampled after HS/LL and resuscitation. The means of 5 determinations ± SD are shown, except for Saline (mean of 7 determinations ± SD). Asterisks atop “Post” bars indicate statistical differences from corresponding Pre values by Mann-Whitney U test; asterisks above horizontal brackets refer to statistical differences between groups by Kruskal-Wallis test with Dunn’s post-tests. In both instances: *, $p < 0.05$, **, $p < 0.01$, ***, $p < 0.001$.

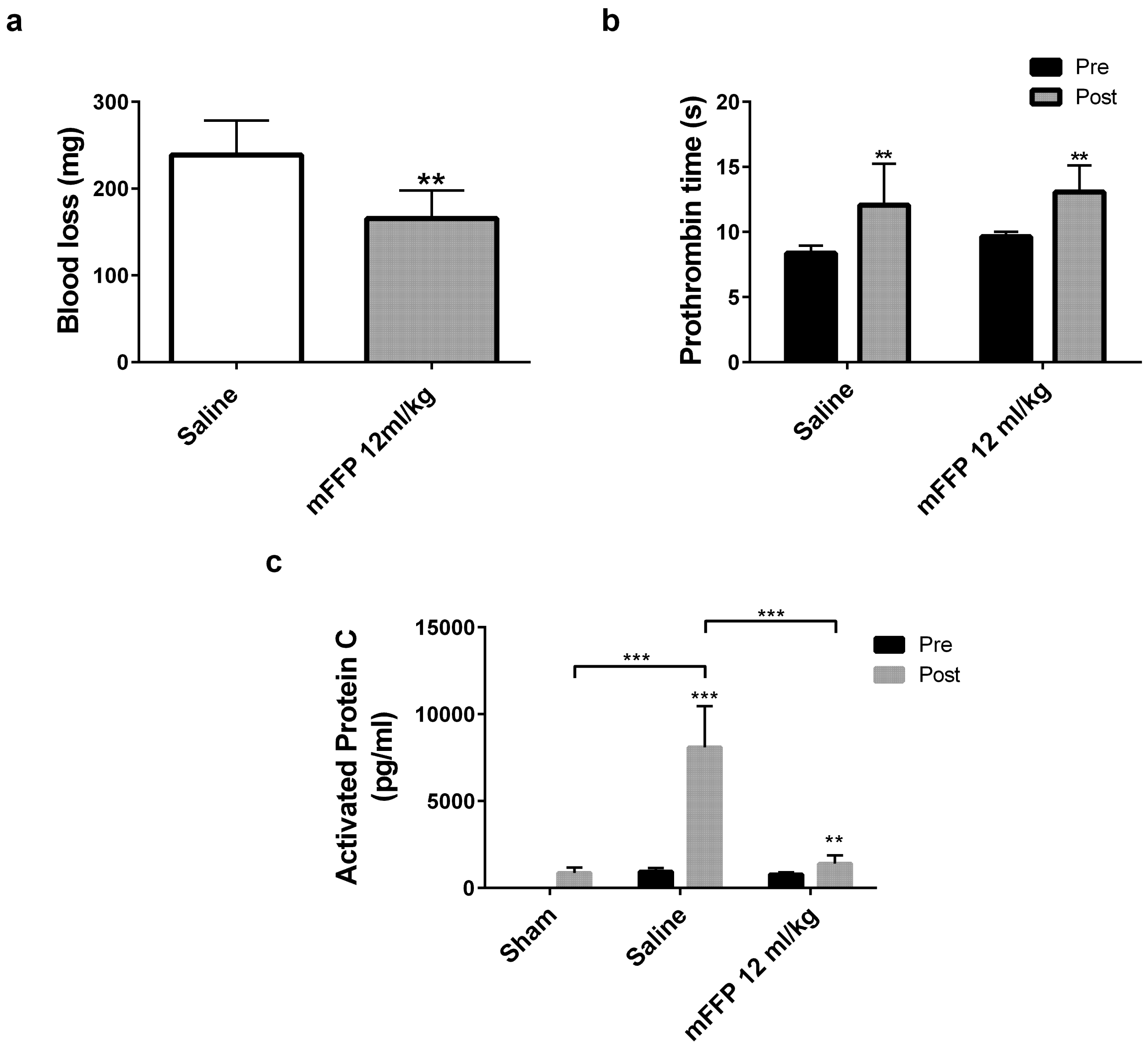

Supplementary Figure 5. Blood losses and clotting times in HS mice resuscitated with saline or 12 ml/kg murine FFP. Panel **a**, mice were subjected to hemorrhagic shock for 60 minutes and then resuscitated with saline (white bars) or murine FFP (mFFP, grey bars) and shed blood was weighed after liver laceration. Panel **b**, Prothrombin Times were determined for plasma from saline-treated mice mouse plasma drawn before shock (Pre, black bars) or after shock and resuscitation (Post, grey bars). Panel **c**, as in **b** but Activated Partial Thromboplastin Times were

determined. Bars show the mean of 6 determinations ± SD (Sham and mFFP 12 ml/kg) or the mean of 8 determinations ± SD (Saline). Asterisks above bars refer to statistical differences by Mann-Whitney U test and asterisks above horizontal brackets refer to statistical differences between groups by Kruskal-Wallis test with Dunn's post-tests (versus saline in **a** and versus Pre and Post in **b** and **c**, *, $p < 0.05$, **, $p < 0.01$, ***, $p < 0.001$).

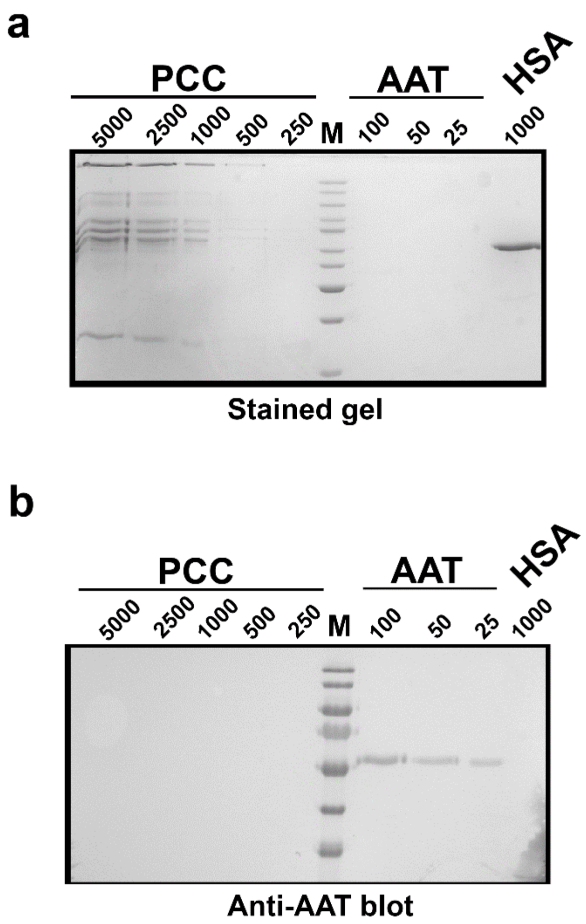

Supplementary Figure 6. Electrophoretic and immunological characterization of PCC. Panel **a** shows a 10% SDS-polyacrylamide gel electrophoresed under reducing conditions and stained with Coomassie Brilliant Blue. PCC or purified AAT or purified HSA (with amounts of protein, in ng, identified above the lanes) were electrophoresed. M, molecular mass markers (kDa): 200; 150; 120; 100; 80; 70; 60; 50; 40; 30; and 25. Panel **b** shows a gel replica of that shown in A, but transferred to nitrocellulose and probed with anti-AAT antibodies as an immunoblot. M, pre-stained molecular mass markers (kDa): 180; 130; 100; 70; 55; 40; 35; and 25.

Tables

Supplementary Table 1: Cytokine ratios in plasma of mice resuscitated with different agents

**Cytokine**	**mFFP Pre/NMP**	**mFFP Post/Pre**	**PCC (14.3 IU/kg) Post/Pre**	**Fgn (140 mg/kg) Post/Pre**
BLC	2.4	1.5	2.6	2.0
C5/C5a	4.3	1.8	1.0	1.7
G-CSF	2.4	4.0	7.7	2.4
GM-CSF	2.4	1.1	2.1	1.4
I-309	2.2	1.0	1.4	1.4
Eotaxin	3.1	0.7	1.1	0.8
sICAM-1	***7.0***	0.9	0.9	0.9
IFN-γ	3.9	0.9	0.8	1.0
IL-1α	1.8	0.5	0.7	0.8
IL-1β	3.7	0.7	0.5	0.8
IL-1ra	4.4	0.5	0.6	0.9
IL-2	4.9	0.7	0.3	0.9
IL-3	4.1	0.8	1.3	2.1
IL-4	4.0	1.4	1.3	2.0
IL-5	4.2	1.5	2.9	1.8
**IL-6**	3.8	**70.8**	**124.5**	**33.7**
IL-7	2.9	1.1	1.2	1.3
**IL-10**	3.9	**28.6**	**53.5**	**17.9**
IL-13	***6.1***	0.8	0.8	1.3
IL-12 p70	4.4	0.7	0.6	1.1
IL-16	3.2	1.5	1.5	1.4
IL-17	4.5	0.7	0.6	0.9
IL-23	4.6	0.5	0.5	0.7
IL-27	4.4	0.5	0.4	0.9
IP-10	2.6	2.0	6.8	3.3
I-TAC	3.0	1.2	1.8	2.1
KC	***5.5***	**80.2**	**82.2**	**42.0**
M-CSF	2.8	1.1	1.0	1.3
**JE**	3.1	**14.0**	**21.8**	**3.6**
MCP-5	4.7	2.7	2.2	1.1
MIG	4.7	1.3	11.0	1.5
MIP-1α	4.1	2.6	2.7	1.3

Supplementary Table 1, continued

**Cytokine**	**mFFP Pre/NMP**	**mFFP Post/Pre**	**PCC (14.3 IU/kg) Post/Pre**	**Fgn (140 mg/kg) Post/Pre**
MIP-1β	***5.1***	4.9	8.1	1.7
**MIP-2**	4.6	**11.1**	**30.2**	**8.1**
**RANTES**	***6.8***	0.5	0.5	0.8
SDF-1	4.9	0.6	0.9	1.0
TARC	2.5	0.9	1.8	2.2
TIMP-1	1.4	1.7	1.5	2.5
**TNF-α**	2.3	**21.9**	**26.2**	**11.4**
TREM-1	2.4	1.3	1.9	2.1

Cytokine names that are bolded and italicized highlight those with a pre-MMP/NMP ratio > 5.

Those that are bolded highlight cytokines elevated across all 3 treatments by a factor > 3.6.
